# Hindlimb spasticity after unilateral motor cortex lesion in rats is reduced by contralateral nerve root transfer

**DOI:** 10.1042/BSR20160412

**Published:** 2016-12-23

**Authors:** Haiyang Zong, Fenfen Ma, Laiyin Zhang, Huiping Lu, Jingru Gong, Min Cai, Haodong Lin, Yizhun Zhu, Chunlin Hou

**Affiliations:** * Department of Orthopedic Surgery, Changzheng Hospital, The Second Military Medical University, Shanghai 200003, China; † Department of Pharmacy, Pudong Hospital, Fudan University, Shanghai 201399, China; ‡ Department of Pharmacy, Linyi People's Hospital, Linyi 276000, China; § Department of Pharmacology, School of Pharmacy, Fudan University, Shanghai 201203, China; ║ State Key Laboratory of Quality Research in Chinese Medicine, Macau University of Science and Technology, Avenida Wai Long, Taipa Macau 999078, China

**Keywords:** hindlimb, motor cortex, nerve transfer, spasticity

## Abstract

Lower extremity spasticity is a common sequela among patients with acquired brain injury. The optimum treatment remains controversial. The aim of our study was to test the feasibility and effectiveness of contralateral nerve root transfer in reducing post stroke spasticity of the affected hindlimb muscles in rats. In our study, we for the first time created a novel animal hindlimb spastic hemiplegia model in rats with photothrombotic lesion of unilateral motor cortex and we established a novel surgical procedure in reducing motor cortex lesion-induced hindlimb spastic hemiplegia in rats. Thirty six rats were randomized into three groups. In group A, rats received sham operation. In group B, rats underwent unilateral hindlimb motor cortex lesion. In group C, rats underwent unilateral hindlimb cortex lesion followed by contralateral L4 ventral root transfer to L5 ventral root of the affected side. Footprint analysis, Hoffmann reflex (H-reflex), cholera toxin subunit B (CTB) retrograde tracing of gastrocnemius muscle (GM) motoneurons and immunofluorescent staining of vesicle glutamate transporter 1 (VGLUT1) on CTB-labelled motoneurons were used to assess spasticity of the affected hindlimb. Sixteen weeks postoperatively, toe spread and stride length recovered significantly in group C compared with group B (*P*<0.001). *H*_max_ (H-wave maximum amplitude)/*M*_max_ (M-wave maximum amplitude) ratio of gastrocnemius and plantaris muscles (PMs) significantly reduced in group C (*P*<0.01). Average VGLUT1 positive boutons per CTB-labelled motoneurons significantly reduced in group C (*P*<0.001). We demonstrated for the first time that contralateral L4 ventral root transfer to L5 ventral root of the affected side was effective in relieving unilateral motor cortex lesion-induced hindlimb spasticity in rats. Our data indicated that this could be an alternative treatment for unilateral lower extremity spasticity after brain injury. Therefore, contralateral neurotization may exert a potential therapeutic candidate to improve the function of lower extremity in patients with spastic hemiplegia.

## Abstract (precis)

Transfer of nerve root from normal side to the spastic side could reduce unilateral motor cortex lesion-induced contralateral hindlimb spasticity in rats.

## INTRODUCTION

Acquired brain injury is a major cause of long-term disability in adults. Spasticity is one of the most common sequelae to stroke or traumatic brain injury survivors. Approximately 30% patients develop spasticity few weeks after stroke [1]. Patients with spasticity showed a lower quality of life compared with patients without this symptom [2]. Furthermore, the direct cost of stroke survivors with spasticity increased 4-fold compared with that of patients without spasticity up to 1 year after stroke [3]. A variety of therapeutic methods have been introduced to manage spasticity of the lower extremities. Conservative treatments such as oral medications, selective nerve blocks, botulinum toxin injections and functional electrical stimulations are effective in spasticity relieving and deformity prevention. Surgery is indicated when the non-operative approaches fail. Selective peripheral neurotomy can be used to relieve excessive spasticity [4]. Tendon lengthening may be required when soft tissue contracture is present [5]. However, the optimal treatment of spasticity remains undetermined. For brachial plexus injury, contralateral C7 nerve root transfer has been widely used with no significant impairment of the healthy upper extremity [6,7], presumably due to the compensatory function of adjacent nerve roots. Similar phenomenon has been observed in the lumbosacral plexus [8,9]. We, therefore, hypothesized that contralateral lumbar nerve root transfer to the affected side might reduce spasticity of the hindlimb muscles after a unilateral motor cortex lesion.

## MATERIALS AND METHODS

### Animals

Thirty-six adult male Sprague–Dawley rats weighing 200–240 g were used in the present study. All animals were provided by Fudan University animal lab. Throughout the experiment, animals were housed in groups of three to four animals per cage with food and water ad libitum and maintained under 12-h light–dark cycle. Animals were randomized into three groups with 12 rats in each group. Group A received sham operation. Group B received a photothrombotic cortex lesion without treatment. Group C received a photothrombotic cortex lesion followed by contralateral L4 ventral root transfer. The present study was approved by the Fudan University Animal Care and Use Committee. The animals were treated according to the Animal Care Guidelines of the National Bureau of Health.

### Creation of a motor cortex lesion

Unilateral motor cortex lesion was induced by photothrombosis as previously described with some modification [10]. Rats were anaesthetized with 10% chloral hydrate (300 mg/Kg body weight; Sinopharm Chemical Reagent) through intraperitoneal injection. Body temperature was maintained at 37°C ± 0.5 using a heating pad. The rats were restrained in a stereotaxic apparatus (ST-5ND-C, Chengdu Instrument factory). Under sterile conditions, a midline incision was made on the vertex of the scalp to expose the calvarial bone. Erythrosine B (20 mg/kg body weight, Sigma–Aldrich) was injected via the lateral tail vein. A beam of light from a fibre optic bundle of a cold light source (FC-532, SFOLT) with a wavelength of 532 nm and power of 170 mW was focused on the exposed skull for 10 min. The light beam illumination was targeted over the left cortical region corresponding to the right hindlimb (bregma +0.9 to −2.4, midline −1.0 to −4.9) for 10 min [11]. The scalp incision was then closed. In group A, rats received the similar procedure without erythrosine B injection or light illumination.

### Nerve root transfer

One week after the creation of a photothrombotic motor cortex lesion, rats in group C were anaesthetized with 10% chloral hydrate as described above and fixed in a prone position. The back was shaved and sterilized. Through a dorsal midline incision, the L3 to L6 lamina was removed and dura was opened. The left (normal side) distal L4 and the right (affected side) proximal L5 ventral roots were surgically divided. The proximal end of the left L4 root and the distal end of the right L5 root were coapted with 12-0 nylon sutures (Chenghe Microscopic instrument factory) under an operating microscope (SXP-1B, medical optical instrument factory). The distal L4 ventral root and proximal L5 ventral root were buried in the paravertebral muscle to prevent nerve regeneration. The wound was closed in layers. Rats in groups A and B received the similar procedure without opening the dura and nerve transfer. Postoperatively, animals received penicillin (200000 units/day) via intraperitoneal injection for 3 days. The animals were numbered after surgery to allow observation and measurements in a blinded manner.

### Footprint analysis

Footprint analysis was conducted 16 weeks after nerve transfer surgery (*n*=6 for each group). Nerve regenerates at 1mm/day. The distance from the lumber (L) spine to the plantaris muscles (PMs) is approximately 10 cm. Therefore, we chose 16 weeks to assess function when nerve regeneration should have been completed. Rats were tested in a gangway (15 cm wide, 80 cm long) with a black shelter at the end. The hindlimbs were coated with black ink, and then the rats were let to walk along the gangway on a piece of white paper. The footprints left on the paper were then analysed. The distance between the first and fifth toes (toe spread) and the stride length were measured from each animal as previously described [12]. A significant reduction in toe spread and/or stride length of the hindlimb contralateral to the infarct compared with that of ipsilateral hindlimb was considered as spasticity [13]. Data were expressed as percentage of change from the control group.

### H-reflex recording

Hoffmann reflex (H-reflex) recordings were performed one day after footprint analysis as described previously [14]. Rats were anaesthetized with ketamine (200 mg/kg i.p., Hengrui medicine). An additional one-third dosage of initial dose was given, if needed, to eliminate voluntary movements. All four limbs were fixed on a platform and their positions were adjusted to avoid unnecessary pressure and stretch. The right hindlimb was shaved and sterilized. A pair of receiving electrodes was inserted into the PM between the third and fourth metatarsal bones. A stimulation electrode was percutaneously inserted behind the medial malleolus. All the electrodes were connected to a four-channel electrophysiology apparatus (Esaote Phasis, Esaote Biomedica). The nerve was stimulated with a single pulse (0.2 ms, 0.1 Hz) at a starting current of 0.1 mA with 0.2 mA increments until a maximum current was reached. H-wave and M-wave thresholds were determined and the *H*_max_ (H-wave maximum amplitude) /*M*_max_ (M-wave maximum amplitude) ratio was calculated. After a 15-min interval, the medial gastrocnemius muscle (GM) was stimulated in a similar fashion.

### Retrograde tracing of the motoneurons of the gastrocnemius muscle

Cholera toxin subunit B (CTB), a high sensitive tracer, was used to label the motoneurons of the right GM. Fifteen weeks after nerve transfer, the right GM was surgically exposed via a posterior midline incision under anaesthesia. A total of 20 μl of 1% CTB labelled with FITC (Sigma–Aldrich) was injected in the right GM at four points in each rat (*n*=6 for groups A and B and *n*=5 for group C). The wound was irrigated with saline and the incision was closed.

### Tissue processing and immunofluorescent staining

One week after CTB injection, rats were given an overdose of 10% chloral hydrate and transcardially perfused with 300 ml of normal saline followed by 300 ml of 4% paraformaldehyde solution in 0.1 M PBS (pH 7.4). The spinal cord segments (L4, L5 and L6) were removed, and immersed in 4% paraformaldehyde overnight at 4°C, followed by 30% sucrose in 0.1 M phosphate buffer (PB) at 4°C overnight. The spinal cords were sectioned coronally at 40 μm thick with a frozen sliding microtome (Microm HM505E). Every tenth section was chosen for immunofluorescent staining. Five sections were obtained from each spinal cord segment. Sections were blocked with 0.01 M PBS solution (pH 7.4) containing 0.3% Triton X-100, 3% BSA and 5% normal donkey serum at room temperature for 2 h. Then, the sections were incubated with vesicle glutamate transporter 1 (VGLUT1) (1:1000, Chemicon) diluted in primary antibody dilution buffer at 4°C overnight. The primary antibody was omitted for negative control. After the primary antibody incubation, all sections were washed in 0.01 M PBS and incubated with Alexa Fluor 594 donkey anti-guinea pig secondary antibody solution (1:200, Sigma–Aldrich) at 37°C for 1 h. The sections were mounted on glass slides coated with anti-fade mounting medium (Guge Biological Technology).

### Quantification of VGLUT1 boutons on CTB-labelled motoneurons

CTB-labelled motoneurons of each section were observed under a fluorescence microscope (DWLB2, Leica). The numbers of VGLUT1 immunoreactive synapses on CTB-labelled motoneurons were quantified. CTB-labelled motoneurons and VGLUT1 positive boutons from each group were scanned by confocal microscopy (TCS SP8, Leica). Inclusion criteria as described by Tan et al. [15] are: complete labelling of soma and proximal dendritic arbour, no overlap with adjacent motoneurons. Under 63 oil-immersion lens, several optical sections at 0.5 μm separation photograph were acquired from the CTB-labelled motoneurons. In all sections, the motoneurons were scanned with a 488 nm wavelength, and then the synapses were detected at 594 nm wavelength. All optical sections were reconstructed at *z*-stack. The number of VGLUT1 synapses on each motoneuron was counted and averaged.

### Muscle mass and cross-sectional area of muscle fibres from the left extensor digitorum longus muscles

After electromyogram examination, the extensor digitorum longus (EDL) muscles were harvested from the left hindlimbs. The muscle was weighed using an electronic balance (scale interval: 0.1 mg, R200D, Sartorius). Then, muscle tissues were immersed overnight in 4% paraformaldehyde, subsequently dehydrated with graded ethanol, embedded in paraffin and cut into transverse sections at a thickness of 5 μm. Sections were stained with haematoxylin and eosin (HE) and photographed under a microscope (DWLB2, Leica). For each section, cross-sectional area of muscle fibres were taken from five random fields and measured with a Leica QWin software package (Q550 IW Image Analysis System, Leica Imaging Systems).

### Nissl staining of the brain sections

The brains were removed in rats after harvesting the spinal cord. Tissues were immersed in 4% paraformaldehyde, dehydrated with 30% sucrose solution and cut at 40 μm thickness sections. Then, the sections were stained with Nissl staining method [16] and photographed with a microscope (DWLB2, Leica).

### Histological and ultrastructural examination of the regenerative nerve

The nerve segments proximal and distal to the anastomosis from four rats in group C were harvested and fixed in 0.1 M phosphate-buffered 4% paraformaldehyde overnight, dehydrated with different concentrations of ethanol and embedded in the paraffin. The tissues were cut into 1 μm thickness sections. Five sections from the proximal segment and five sections from the distal segment were obtained and stained with 0.5% toluidine blue (Borong Biotechnology). Sections were photographed under a light microscope (DWLB2, Leica). Leica QWin software package (Q550 IW Image Analysis System, Leica Imaging Systems) was used for nerve fibre counting. The total numbers of axons in the proximal and distal segments were averaged. The nerve coaptation sites were fixed in 2.5% glutaraldehyde, dehydrated in ethanol and propanone and infiltrated with resin. Then, tissue were cut into ultrathin sections and stained with 3% uranyl acetate–lead citrate. The ultrastructure of regenerative nerve axons were photographed with the use of TEM (CM l20, Philips).

### Statistical analysis

Data were expressed as means ± S.D. Normal data distribution and homogeneity variance were confirmed. Differences between groups were compared with one-way ANOVA followed by post hoc comparisons of Tukey's test. *P*-values <0.05 were considered statistically significant using SPSS 17.0 Statistical Software (SPSS).

## RESULTS

### Cerebral lesions

**Figure 1:**
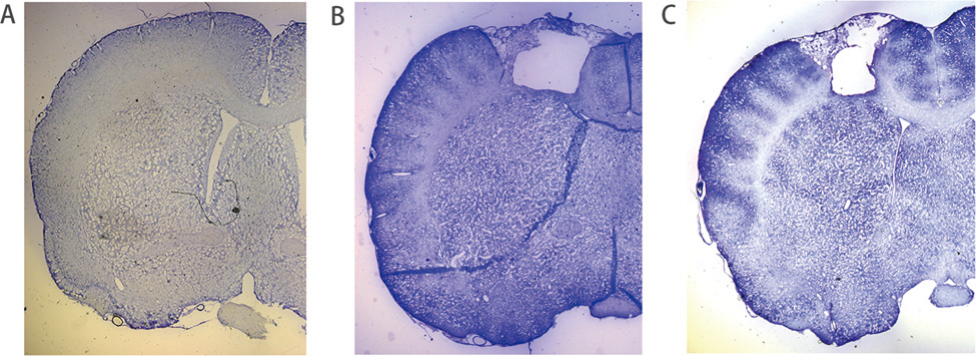
Histological changes of the left motor cortex in three groups Photothrombotic cortical lesion in the left hindlimb motor cortex (Nissl stain, magnification ×25). (**A**) Nissl-stained coronal section from group A. (**B** and **C**) Nissl-stained coronal sections from groups B and C, the left motor cortex showed specific lesion.

One rat in group C developed back wound infection one week after nerve transfer and, therefore, was excluded from the study. Cerebral lesions were successfully created in all animals in groups B and C and were confirmed by Nissl staining (Figures 1B and 1C). No infract was seen in specimens from group A animals (Figure 1A).

### Footprint analysis

Toe spread and stride length measurements are summarized in Table 1. Both parameters from the right hindlimbs 16 weeks following a cerebral lesion (groups B) were significantly reduced compared with that in group A (*P*<0.001). After nerve root transfer (group C), the right hindlimb toe spread and stride length were significantly greater compared with that in group B (*P*<0.001), suggesting some relief of spasticity. The average body weight (589.8±8.5, 583.8±10.8, 577.3±9.1 g in groups A, B and C respectively) was not significantly different among the three groups (*P*>0.05).

**Table 1:** Footprint analysis of each group 16 weeks postoperatively (Data are expressed as percentage of change from average values of group A, mean ± S.D.)

Parameter	Group A	Group B	Group C
Left toe spread	100.17±1.39	99.84±2.02	100.236±1.20
Right toe spread	100.02±2.01	88.41±2.20	92.07±2.18***
Left stride length	100.40±3.57	89.76±2.74	90.64±3.23
Right stride length	100.11±3.56	81.94±3.91	89.31±2.57***

***P<0.001, compared with group B.

### *H*_max_/*M*_max_ of the right medial gastrocnemius and plantaris muscles

**Figure 2:**
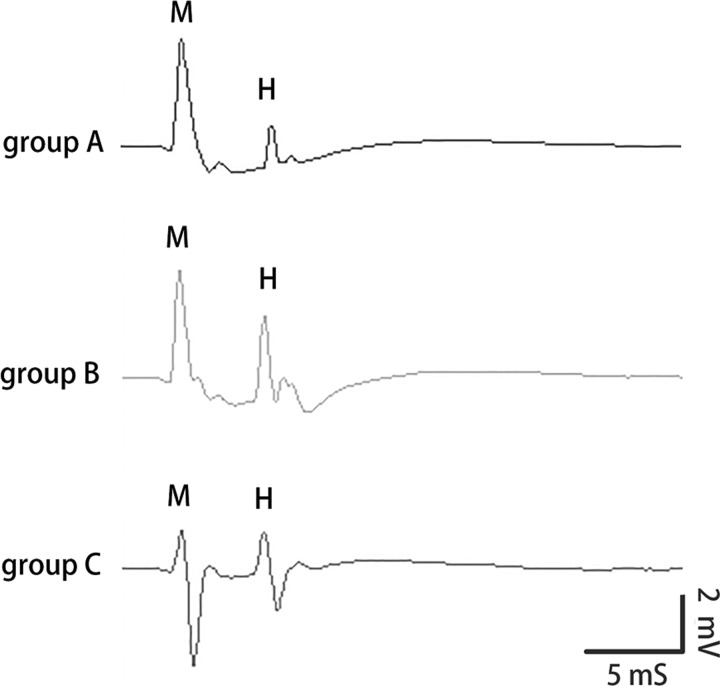
Representative electromyography results of H-reflex in three groups M-wave and H-wave from the PMs in three groups. In group B, the maximum H-reflex amplitude increased after motor cortex lesion. In group C, the maximum amplitude of H-reflex decreased after contralateral nerve root transfer.

The maximum H-wave amplitude of the right medial gastrocnemius and PMs was significantly increased in group B compared with that in group A (*P*<0.001). There was a significant reduction in these measurements in group C compared with that in group B (Figure 2). There were no significant changes of M-wave amplitude in the right medial gastrocnemius and PMs in groups B and C compared with that in group A (*P*>0.05). The *H*_max_/*M*_max_ ratio of the right medial gastrocnemius and PMs in group B was significantly higher than that in group A (*P*<0.001). The *H*_max_/*M*_max_ ratio in group C was significantly lower compared with that in group B (Table 2).

**Table 2:** M-wave, *H*_max_ and *H*_max_/*M*_max_ ratio in the right gastrocnemius and PMs (mean ± S.D., mV)

Muscle	Parameter	Group A	Group B	Group C
PM	*M*_max_ (mV)	8.3±0.5	8.2±0.6	7.7±0.4
	*H*_max_ (mV)	1.7±0.1	3.3±0.4	2.6±0.2***
	*H*_max_/*M*_max_ ratio (%)	21.0±2.0	40.8±2.1	33.5±1.9***
GM	*M*_max_ (mV)	42.3±3.4	43.9±3.5	39.6±2.9
	*H*_max_ (mV)	3.5±0.5	7.8±0.90	5.63±0.9**
	*H*_max_/*M*_max_ ratio (%)	8.3±1.5	18.5±1.9	14.2±1.5**

**P<0.01, ***P<0.001, compared with group B.

### Quantification of VGLUT1 boutons on CTB-labelled motorneurons

**Figure 3:**
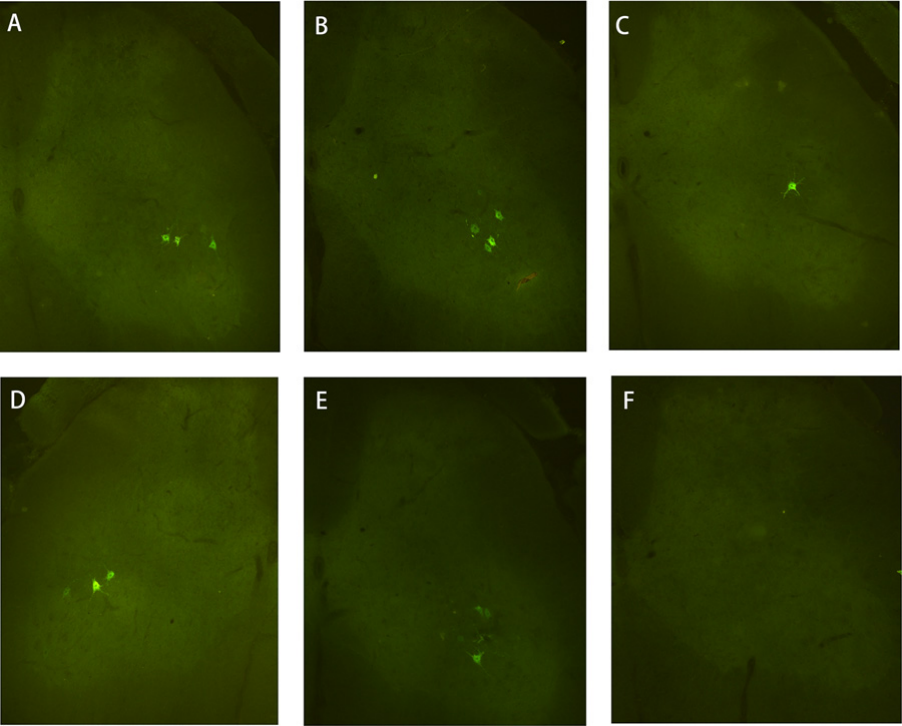
Photographs of CTB labeled motoneurons in three groups Retrograde labelled motoneruons after injection of CTB in the right GM (green, CTB-labelled motoneurons, magnification ×100). In the non-neurotization groups, CTB-labelled motoneurons were located in the right ventral horn of L4 (**A**), L5 (**B**) and L6 (**C**) spinal cord. In group C, CTB-labelled motoneurons were observed in left (**D**) and right (**E**) side of L4 spinal cord but absent from the right L5 ventral horn (**F**).

**Figure 4:**
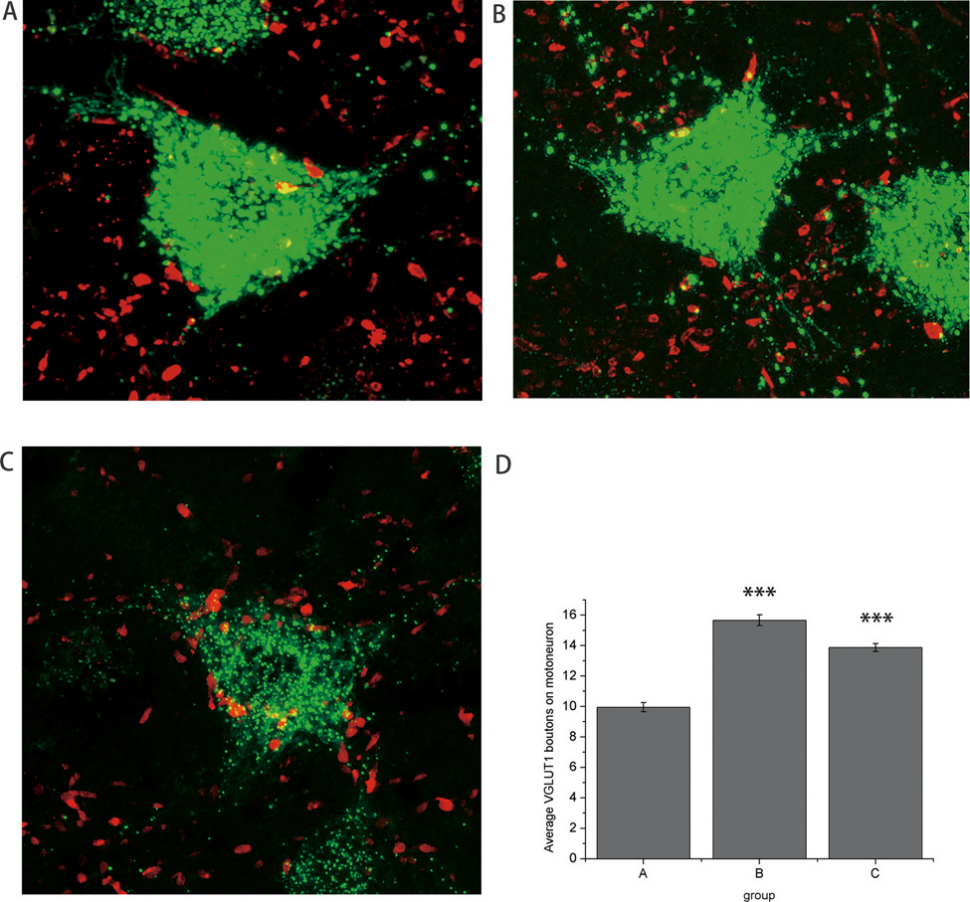
Quantification of VGLUT1 boutons on CTB labeled motoneurons VGLUT1 positive boutons on CTB-labelled motoneurons. Confocal *z*-stack images from three groups showed CTB-labelled motoneurons (green) and VGLUT1 immunopositive boutons (red). Number of VGLUT1 positive boutons on motoneurons increased in the right side of L5 spinal cord in group B (**C**) compared with right L5 spinal cord in group A (**A**) and left L4 spinal cord in group C (**B**) (immunofluorescent stain, magnification ×630). (**D**) The average number of VGLUT1 positive boutons on motoneurons significantly increased in group B compared with that in group A (***P<0.001). After contralateral nerve root transfer, the average number of VGLUT1 positive boutons on motoneurons significantly reduced in group C compared with that in group B (***P<0.001).

The CTB retrograde labelled motoneurons were identified in each spinal cord segment from the right ventral horn in groups A and B. In group C, the labelled motoneurons were found on both sides of L4 ventral horn and the right side of L6 ventral horn, but not in the L5 spinal cord segment (Figure 3). The average number of VGLUT1 positive boutons on CTB-labelled motoneurons was significantly increased in group B (Figure 4C) compared with that in group A (Figure 4A, *P*<0.001). In group C, the number of VGLUT1 positive boutons on CTB-labelled motoneurons in the right ventral horn was also increased, but not in the left L4 ventral horn (Figure 4B). Thus, the average number of VGLUT1 on CTB-labelled motoneurons was significantly reduced in group C compared with group B (Figure 4D, *P*<0.001).

### Muscle mass and cross-sectional area of muscle fibres from the left EDL muscles

**Figure 5:**
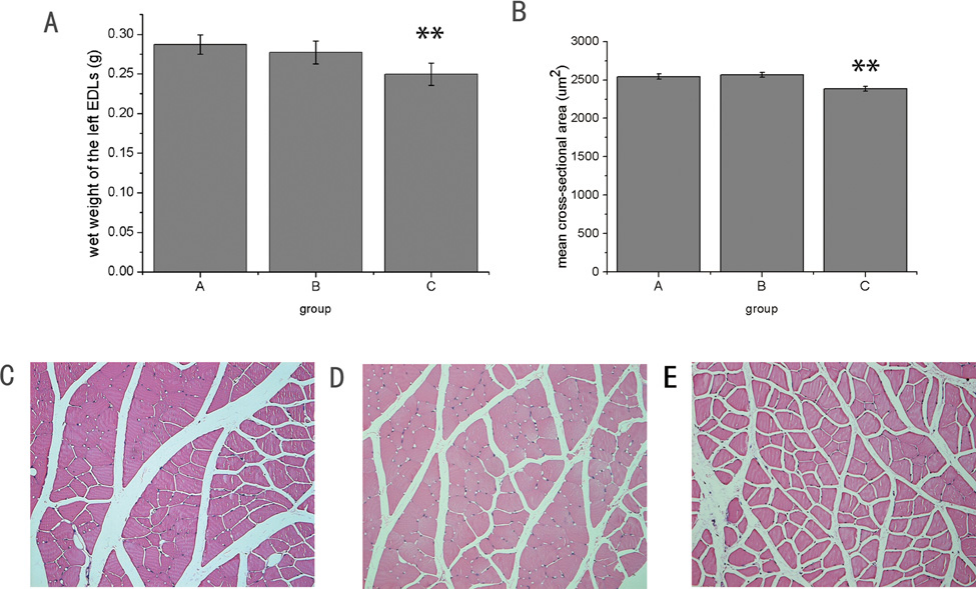
Wet weight and cross-sectional area of the left EDL muscles (**A**) Wet weight of the left EDL muscle in group C was reduced (**P<0.01) compared that in group A and B. (**B**) Mean cross sectional-area of muscle fibers from group C was smaller than that in group A and B. Photographs of cross-sections from group A (**C**), group B (**D**) and group C (**E**) (HE stain, magnification ×200).

There were no significant differences of the mean wet weight of the left EDL muscle between group A and group B (*P*>0.05) (Figure 5A). The mean wet weight of the EDL muscle in group C was significantly lower (*P*<0.01) compared with that in groups A and B (Figure 5A). The cross-sectional area of muscle fibres from the left EDL muscles in group C was significantly decreased (*P*<0.01) compared with that in groups A and B (Figure 5B). Histologic examination of muscle fibres from groups A (Figure 5C), B (Figure 5D) and C (Figure 5E) were shown.

### Histological and ultrastructure of the regenerative nerve

**Figure 6:**
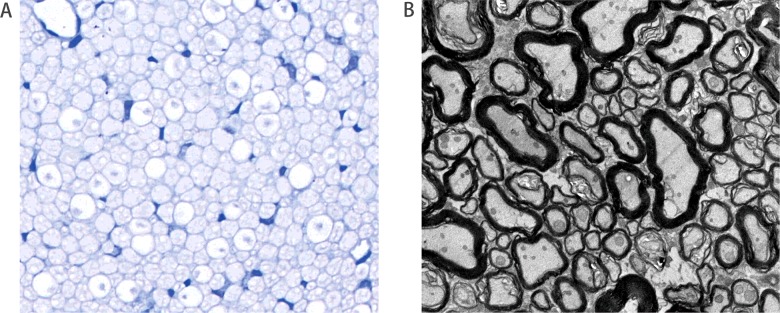
Histological and ultrastructure of the regenerative nerve (**A**) Photograph of the cross-section of the regenerated nerve distal to coaptation site in group C (toluidine blue stain, magnification ×640). There was a high density of myelinated axons of the regenerative nerve. (**B**) Electron micrograph of the regenerative nerve at the coaptation site in group C (lead citrate stain, magnification ×3000). The regenerated nerve with myelinated and unmyelinated fibres was observed.

Histological examination of the regenerative nerves showed prominent axonal regeneration in all specimens (Figure 6A). The distal-to-proximal axon regeneration ratio of the nerve root was 69.61±4.09%. Ultrastructure of the neurorrhaphy site showed regenerated myelinated and unmyelinated axons, suggesting regrowth of nerve fibres (Figure 6B).

## DISCUSSION

Acquired brain injury-related disability in the lower limbs is thought to be the result of disruption of the descending pathways. The impairments are characterized by spasticity, muscle overactivity, contractures, muscle weakness and loss of dexterity [17,18]. Many therapeutic modalities have been introduced but results vary and side effects could be prohibitive [19]. Based on previous experience of contralateral C7 nerve root transfer for the treatment of upper extremity paralysis after central neurologic injury [20], we hypothesized that contralateral lumbar nerve root transfer might reduce lower limb spasticity following the creation of a motor cortical lesion.

In the present study, we successfully established an animal model of photothrombotic motor cortex lesion to generate contralateral hindlimb spasticity in rats. Then, the contralateral L4 nerve root was transferred to the ipsilateral L5 root. Spasticity of the hindlimb muscles in the affected side was evaluated by locomotor function, electrophysiology and immunohistochemical analysis [13,21]. The results showed that, sixteen weeks postoperatively, toe spread and stride length were recovered significantly in group C compared with group B. *H*_max_/*M*_max_ ratio of gastrocnemius and PMs was reduced greatly in group C, whereas average VGLUT1 positive boutons per CTB-labelled motoneuron decreased substantially in group C. The results demonstrated that hindlimb spasticity induced by unilateral motor cortex lesion was relieved after the contralateral L4 ventral root transferred to L5 ventral root in rats.

The present study suggests that contralateral nerve root may be considered as a potential donor nerve to reduce hindlimb spasticity after unilateral motor cortex lesion in humans. There were two possible reasons for this new surgical procedure to relieve hindlimb spasticity after unilateral motor cortex lesion. Firstly, selective dorsal rhizotomy was used to treat the cerebral palsy for decades [22]. The basis of dorsal rhizotomy is that afferent terminals input a net excitatory effect on the efferent output via the interneuron pool. Section the dorsal root has been proved to reduce the amount of afferent excitation and thereby relieve spasticity [23]. In our procedure, severing the L5 ventral root in the affected side indirectly isolated a proportion of excited afferent fibres. As a result, spasticity of the affected hindlimb muscles would be relaxed. Secondly, when the contralateral L4 efferent fibres reinnervated the affected muscles in the spastic side, these normal functioned fibres reinnervated the contralateral hindlimb muscles, which reinforced active control of the spastic hindlimb from the uninjured hemisphere. This was supported by our findings of the presence of CTB retrograde labelled motoneurons in the left L4 ventral horn and histological evidence of nerve regeneration. The regenerated motor nerve reinnervated the affected hindlimb muscles, which was also supported by our results that the M-wave amplitude was not significantly reduced in the right hindimb muscles after nerve root transfer, whereas the H-wave amplitude was reduced, as a result, spasticity of the hindlimb muscles relieved. As we know, hindlimb muscles are dominated by multiple nerve roots. Reinnervation by one nerve root from the unaffected side only replaced part of nerve fibres of the affected muscles. In addition, the distal-to-proximal axon regeneration ratio of the nerve root was approximately 70 percent, so hindlimb muscle spasticity was relieved partially in our study.

Our previous study has revealed that severing the L6 nerve root of the sacral (S) plexus in rhesus monkey does not impair the lower limb function that was assessed via muscle mass, electrophysiology, muscle fibre cross-sectional area and ultrastructure of the target muscles [24]. Recently, Zhu et al. [25] reported no long-term impairment had been found in the donor lower limb in a clinical trial by transferring normal S1 nerve root to repair contralateral lumbosacral plexus avulsion. However, we observed atrophy of the EDL muscles after severing the L4 ventral root in rats as indicated by muscle mass and muscle fibre cross-sectional area. It should be pointed out that the S plexus are composed of five nerve roots in human, sectioned one nerve root in the middle of the plexuses only denervated a small proportion of nerve fibres from their target muscles. Therefore, the partial denervated muscles were not significantly impaired. Nevertheless, the rat's sciatic nerves are composed of L4, L5 and L6 nerve roots [26]. The major neural input to the EDL muscle comes through the L4 nerve root, which supplies approximately 60–80% of the innervation [27]. Anatomical differences between rats and primates may explain the atrophy of the EDL muscles in rats after severing the L4 nerve root.

Of course, we pointed out some new issues to be solved in the future. Firstly, spasticity assessment in human mainly based on physical examination such as the Modified Tardieu Scale scored by the degree of muscle tone [28]. In animal models, spasticity assessment was mainly based on H-reflex examined by electromyography, and excitatory of motorneurons in the spinal cord such as VGLUT1 boutons on motoneurons and movement disorders [13–15]. Apparent spasticity behaviour was not observed in rodents previously as well as our study following cortical infarction [29], probably due to slight muscle tone changed in rodents after central nervous system injury, and it's not easy to examined and scaled. This discrepancy needs to be solved in the future. Moreover, as anatomy differences between rats and human, the safety and feasibility of this surgical procedure need to be studied in animals whose S plexus composition is similar to human.

In conclusion, our data indicated that this could be an alternative treatment for unilateral lower extremity spasticity after brain injury. Contralateral L4 ventral nerve root transfer to L5 ventral root on the affected side after unilateral motor cortex lesion to reduce spasticity of the affected hindlimb muscles was feasible and effective in this rat model. Therefore, contralateral nerve root may be used as a donor nerve to reduce hindlimb spasticity after unilateral motor cortex lesion in humans. Contralateral neurotization may exert a potential therapeutic candidate to improve the function of lower extremity in patients with spastic hemiplegia.

## Abbreviations

CTB: cholera toxin subunit B
EDL: extensor digitorum longus
GM: gastrocnemius muscle
HE: haematoxylin and eosin
*H*_max_: H-wave maximum amplitude
H-reflex: Hoffmann reflex
L: lumber
*M*_max_: M-wave maximum amplitude
PB: phosphate buffer
PM: plantaris muscle
S: sacral
VGLUT1: vesicle glutamate transporter 1

## References

[1] Mayer N.H., Esquenazi A. (2003). Muscle overactivity and movement dysfunction in the upper motoneuron syndrome. Phys. Med. Rehabil. Clin. N. Am..

[2] Urban P.P., Wolf T., Uebele M., Marx J.J., Vogt T., Stoeter P., Bauermann T., Weibrich C., Vucurevic G.D., Schneider A., Wissel J. (2010). Occurence and clinical predictors of spasticity after ischemic stroke. Stroke.

[3] Lundström E., Smits A., Borg J., Terént A. (2010). Four-fold increase in direct costs of stroke survivors with spasticity compared with stroke survivors without spasticity: the first year after the event. Stroke.

[4] Decq P., Shin M., Carrillo-Ruiz J. (2004). Surgery in the peripheral nerves for lower limb spasticity. Oper. Tech. Neurosurg..

[5] King B.W., Ruta D.J., Irwin T.A. (2014). Spastic foot and ankle deformities: evaluation and treatment. Foot Ankle Clin..

[6] Gu Y.D., Zhang G.M., Chen D.S., Yan J.G., Cheng X.M., Chen L. (1992). Seventh cervical nerve root transfer from the contralateral healthy side for treatment of brachial plexus root avulsion. J. Hand Surg. Br..

[7] Terzis J.K., Kokkalis Z.T. (2009). Selective contralateral c7 transfer in posttraumatic brachial plexus injuries: a report of 56 cases. Plast. Reconstr. Surg..

[8] Lin H., Chen A., Hou C. (2013). Contralateral L-6 nerve root transfer to repair lumbosacral plexus root avulsion: experimental study in rhesus monkeys. J. Neurosurg..

[9] Lin H., Hou C. (2013). Transfer of normal S1 nerve root to reinnervate atonic bladder due to conus medullaris injury. Muscle Nerve.

[10] Hu X., Johansson I.M., Brännström T., Olsson T., Wester P. (2002). Long-lasting neuronal apoptotic cell death in regions with severe ischemia after photothrombotic ring stroke in rats. Acta Neuropathol..

[11] Seong H.Y., Cho J.Y., Choi B.S., Min J.K., Kim Y.H., Roh S.W., Kim J.H., Jeon S.R. (2014). Analysis on bilateral hindlimb mapping in motor cortex of the rat by an intracortical microstimulation method. J. Korean Med. Sci..

[12] de Medinaceli L., Freed W.J., Wyatt R.J. (1982). An index of the functional condition of rat sciatic nerve based on measurements made from walking tracks. Exp. Neurol..

[13] Meier C., Middelanis J., Wasielewski B., Neuhoff S., Roth-Haerer A., Gantert M., Dinse H.R., Dermietzel R., Jensen A. (2006). Spastic paresis after perinatal brain damage in rats is reduced by human cord blood mononuclear cells. Pediatr. Res..

[14] Kakinohana O., Hefferan M.P., Nakamura S., Kakinohana M., Galik J., Tomori Z., Marsala J., Yaksh T.L., Marsala M. (2006). Development of GABA-sensitive spasticity and rigidity in rats after transient spinal cord ischemia: a qualitative and quantitative electrophysiological and histopathological study. Neuroscience.

[15] Tan A.M., Chakrabarty S., Kimura H., Martin J.H. (2012). Selective corticospinal tract injury in the rat induces primary afferent fiber sprouting in the spinal cord and hyperreflexia. J. Neurosci..

[16] Parent J.M., Valentin V.V., Lowenstein D.H. (2002). Prolonged seizures increase proliferating neuroblasts in the adult rat subventricular zone-olfactory bulb pathway. J. Neurosci..

[17] Esquenazi A., Mayer N.H. (2004). Instrumented assessment of muscle overactivity and spasticity with dynamic polyelectromyographic and motion analysis for treatment planning. Am. J. Phys. Med. Rehab..

[18] Mayer N.H., Esquenazi A., Childers M.K. (1997). Common patterns of clinical motor dysfunction. Muscle Nerve Suppl..

[19] McIntyre A., Lee T., Janzen S., Mays R., Mehta S., Teasell R. (2012). Systematic review of the effectiveness of pharmacological interventions in the treatment of spasticity of the hemiparetic lower extremity more than six months post stroke. Top Stroke Rehabil..

[20] Hua X.Y., Qiu Y.Q., Li T., Zheng M.X., Shen Y.D., Jiang S., Xu J.G., Gu Y.D., Xu W.D. (2015). Contralateral peripheral neurotization for hemiplegic upper extremity after central neurologic injury. Neurosurgery.

[21] Toda T., Ishida K., Kiyama H., Yamashita T., Lee S. (2014). Down-regulation of KCC2 expression and phosphorylation in motoneurons, and increases the number of in primary afferent projections to motoneurons in mice with post-stroke spasticity. PLoS One.

[22] Aquilina K., Graham D., Wimalasundera N. (2015). Selective dorsal rhizotomy: an old treatment re-emerging. Arch. Dis. Child..

[23] Steinbok P. (2007). Selective dorsal rhizotomy for spastic cerebral palsy: a review. Child. Nerv. Syst..

[24] Lin H., Xu Z., Liu Y., Chen A., Hou C. (2012). The effect of severing L6 nerve root of the sacral plexus on lower extremity function: an experimental study in rhesus monkeys. Neurosurgery.

[25] Zhu L., Zhang F., Yang D., Chen A. (2015). The effect of severing a normal S1 nerve root to use for reconstruction of an avulsed contralateral lumbosacral plexus: a pilot study. Bone Joint J..

[26] Schmalbruch H. (1986). Fiber composition of the rat sciatic nerve. Anat. Rec..

[27] Albani M., Lowrie M.B., Vrbová G. (1988). Reorganization of motor units in reinnervated muscles of the rat. J. Neurol. Sci..

[28] Naghdi S., Ansari N.N., Abolhasani H., Mansouri K., Ghotbi N., Hasson S. (2014). Electrophysiological evaluation of the Modified Tardieu Scale (MTS) in assessing poststroke wrist flexor spasticity. NeuroRehabilitation.

[29] Lee S., Toda T., Kiyama H., Yamashita T. (2014). Weakened rate-dependent depression of Hoffmann's reflex and increased motoneuron hyperactivity after motor cortical infarction in mice. Cell Death Dis..

